# Receptor-Mediated Melanoma Targeting with Radiolabeled α-Melanocyte-Stimulating Hormone: Relevance of the Net Charge of the Ligand

**DOI:** 10.3389/fendo.2017.00093

**Published:** 2017-04-26

**Authors:** Jean-Philippe Bapst, Alex N. Eberle

**Affiliations:** ^1^Laboratory of Endocrinology, Department of Biomedicine, University Hospital and University Children’s Hospital, University of Basel, Basel, Switzerland; ^2^Collegium Helveticum, ETH Zurich, Zurich, Switzerland

**Keywords:** melanoma, α-melanocyte-stimulating hormone, radiolabeled peptide, phosphopeptide, tumor targeting, net charge, tissue distribution, kidney toxicity

## Abstract

A majority of melanotic and amelanotic melanomas overexpress melanocortin type 1 receptors (MC1Rs) for α-melanocyte-stimulating hormone. Radiolabeled linear or cyclic analogs of α-MSH have a great potential as diagnostic or therapeutic tools for the management of malignant melanoma. Compounds such as [^111^In]DOTA-NAP-amide exhibit high affinity for the MC1R *in vitro*, good tumor uptake *in vivo*, but they may suffer from relatively high kidney uptake and retention *in vivo*. We have shown previously that the introduction of negative charges into radiolabeled DOTA-NAP-amide peptide analogs may enhance their excretion and reduce kidney retention. To address the question of where to place negative charges within the ligand, we have extended these studies by designing two novel peptides, Ac-Nle-Asp-His-d-Phe-Arg-Trp-Gly-Lys(DOTA)-d-Asp-d-Asp-OH (DOTA-NAP-d-Asp-d-Asp) with three negative charges at the *C*-terminal end (overall net charge of the molecule −2) and DOTA-Gly-Tyr(P)-Nle-Asp-His-d-Phe-Arg-Trp-NH_2_ (DOTA-Phospho-MSH_2-9_) with two negative charges in the *N*-terminal region (net charge −1). The former peptide showed markedly reduced receptor affinity and biological activity by >10-fold compared to DOTA-NAP-amide as reference compound, and the latter peptide displayed similar bioactivity and receptor affinity as the reference compound. The uptake by melanoma tumor tissue of [^111^In]DOTA-Phospho-MSH_2-9_ was 7.33 ± 0.47 %ID/g 4 h after injection, i.e., almost equally high as with [^111^In]DOTA-NAP-amide. The kidney retention was 2.68 ± 0.18 %ID/g 4 h after injection and hence 44% lower than that of [^111^In]DOTA-NAP-amide. Over an observation period from 4 to 48 h, the tumor-to-kidney ratio of [^111^In]DOTA-Phospho-MSH_2-9_ was 35% more favorable than that of the reference compound. In a comparison of DOTA-NAP-d-Asp-d-Asp, DOTA-Phospho-MSH_2-9_ and DOTA-NAP-amide with five previously published analogs of DOTA-NAP-amide that altogether cover a range of peptides with an overall net charge between +2 and −2, we now demonstrate that a net charge of −1, with the extra negative charges preferably placed in the *N*-terminal region, has led to the lowest kidney uptake and retention. Charges of +2 or −2 markedly increased kidney uptake and retention. In conclusion, the novel DOTA-Phospho-MSH_2-9_ may represent a new lead compound for negatively charged linear MC1R ligands that can be further developed into a clinically relevant melanoma targeting radiopeptide.

## Introduction

In the last 30 years, cutaneous malignant melanoma has become one of the most rapidly advancing tumors in the general population and the most commonly occurring tumor among young adults (1); and today, it still continues to increase (2). The prognosis of metastatic melanoma is usually poor. As melanoma cells overexpress membrane-bound melanocortin type 1 receptors (MC1Rs) (3–5), suitable ligands for MC1R are candidates for targeting melanoma with diagnostic or therapeutic radioisotopes (6) or toxin conjugates (7). The natural ligand for MC1R is α-melanocyte-stimulating hormone (α-MSH) for which a number of peptide analogs with increased binding affinity have been synthesized in the past, such as linear [Nle^4^, d-Phe^7^]-α-MSH (NDP-MSH; melanotan I) (8) or cyclic Ac-Nle-*cyclo*[Asp-His-d-Phe-Arg-Trp-Lys]-NH_2_ (melanotan II) (9) and related peptides (10). For melanoma targeting with radioactive metal isotopes, several linear MSH peptides containing chelators for radiometals were developed (11–15). Of these, ^111^In- or ^67/68^Ga-labeled DOTA-NAP-amide exhibited the most promising *in vivo* characteristics as it showed the highest tumor uptake paired with minimal non-tumor tissue uptake, except for the kidneys (13).

High uptake and retention of radioactivity by the kidneys and some other organs is a general problem for most peptide radiopharmaceuticals (16). Several strategies have been developed for radiometal-labeled MSH peptides to reduce kidney uptake such as the use of different chelators (e.g., DOTA, NOTA) and radioisotopes and/or variations in the positioning of the chelator within the peptide molecule (6, 17, 18). Alternatively, introduction of a suitable linker between chelator and peptide may be used (19), or the formation of peptide dimers (20) or cyclic peptides (21, 22). Although dimerization produced some very potent high-affinity compounds *in vitro*, their *in vivo* characteristics were disappointing (20). By contrast, analysis of a number of cyclized α-MSH peptides labeled with ^111^In, ^177^Lu, ^90^Y, ^99m^Tc, or ^188^Re yielded promising lead candidates (23–28) although these compounds in general suffered equally from high kidney uptake. A direct comparison of a linker-extended ^64^Cu-labeled NOTA-NAP-amide peptide with a related cyclic peptide showed that both radiopeptides displayed excellent melanoma targeting, however paired with relatively high kidney uptake (29); the overall radiopharmacological characteristics of the linear peptide were superior in this study.

Another approach to reduce uptake of radioactivity by the kidneys is coinjection of radiopeptides with basic amino acids. As the surface of proximal tubular cells in the kidneys is negatively charged and electrostatic interactions contribute greatly to the uptake of positively charged peptide molecules, basic amino acids may compete with the uptake of radiopeptides and hence reduce it (30). This method was first applied for ^111^In-labeled DTPA-octreotide (31, 32) and ^90^Y-labeled DOTA-[Tyr^3^]-octreotide (32, 33). In particular, coinjection of d-Lys markedly reduced kidney uptake by up to 65% without affecting tumor uptake (32). A lesser reduction was achieved by coinfusion of Lys and Arg with ^188^Re-labeled cyclized MSH (34) or with ^111^In-labeled DOTA-NAP-amide (Froidevaux and Eberle, unpublished). Although successful for some of the radiopeptides, coinjection of basic amino acids only partially solved the problem of kidney toxicity by the accumulated radioactivity.

Following basic reabsorption studies in the kidneys with positively, neutrally, and negatively charged proteins (35, 36), Kok et al. (37) demonstrated that the excretion of succinylated lysozyme is dramatically increased compared to the non-succinylated molecule with six amino groups, suggesting that reduction of the positive net charge of lysozyme had a beneficial effect on urinary excretion. Akizawa et al. (38) confirmed the influence of negative charges on the excretion of ^111^In-labeled DTPA-octreotide peptides containing modifications in position 1 (Asp vs. Phe or Met vs. Lys): the lowest radioactivity counts in the kidneys were found with [^111^In]DTPA-[Asp^1^]-octreotide, whereas the highest were seen with [^111^In]DTPA-[Lys^1^]-octreotide. Less conspicuous data were obtained with [^111^In]DOTA-NAP-amide containing different charges in the *C*-terminal region: although with the DOTA-NAP-amide analog [DOTA-Nle^4^,Asp^5^,d-Phe^7^,Lys^11^(Suc)]-α-MSH_4-11_-carboxylate, a negative charge in the *C*-terminus reduced kidney uptake of the radiopeptide, tumor uptake was also affected, and hence, the tumor-to-kidney ratio was even lower than that of the parent molecule with non-charged *C*-terminal amide (14). By contrast, introduction of a Glu residue at the *N*-terminus of a Re-cyclized DOTA-containing MSH peptide increased the tumor-to-kidney ratio, demonstrating the beneficial effect of an additional negative charge (26).

**Figure 1:**
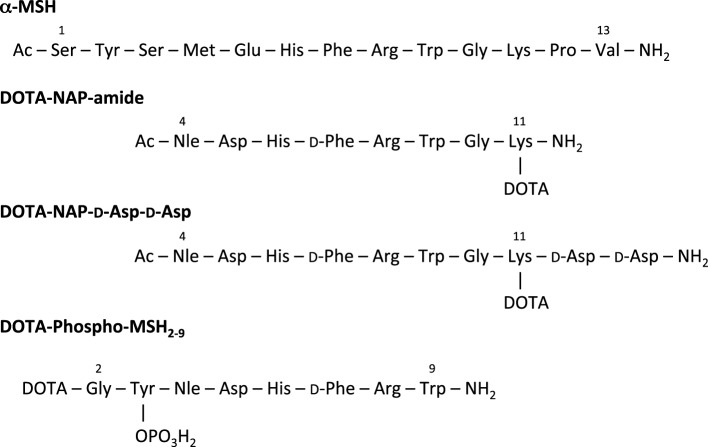
**Amino acid sequence of α-MSH, DOTA-NAP-amide, DOTA-NAP-d-Asp-d-Asp, and DOTA-Phospho-MSH_2-9_**.

To compare the pharmacokinetic properties of linear ^111^In-labeled DOTA-MSH peptide analogs containing a net charge ranging from +2 to −2, we synthesized and biologically characterized two new peptides, Ac-Nle-Asp-His-d-Phe-Arg-Trp-Gly-Lys(DOTA)-d-Asp-d-Asp-OH (abbreviated name: DOTA-NAP-d-Asp-d-Asp) and DOTA-Gly-Tyr(P)-Nle-Asp-His-d-Phe-Arg-Trp-NH_2_ (DOTA-Phospho-MSH_2-9_), and we also included DOTA-NAP-amide as a reference compound in this study (Figure 1). Biodistribution data obtained in tumor-bearing mice were compared with the data of the previously published linear MSH analogs (14). The analysis showed that DOTA-Phospho-MSH_2-9_ yielded the best tumor-to-kidney ratio of all linear MSH peptides so far investigated, demonstrating that an overall net charge of the peptide of −1 with a negatively charged *N*-terminal region resulted in the most favorable biodistribution properties.

## Materials and Methods

### Reagents

α-Melanocyte-stimulating hormone was a gift from the Novartis Institutes of Biomedical Research (Basel, Switzerland). [Nle^4^,D-Phe^7^]-α-MSH (NDP-MSH) was obtained from Bachem (Bubendorf, Switzerland). DOTA-tris(*tert*-butyl ester) (1,4,7,10-tetraazacyclododecane-1,4,7-tris-*tert-*butyl acetate-10-acetic acid) was purchased from Macrocyclics (Dallas, TX, USA), Fmoc-PAL-PEG-PS resin from Applied Biosystems (Rotkreuz, Switzerland), Fmoc-d-Asp(*t*Bu)-TentaGel S AC resin from Rapp-Polymere (Tübingen, Germany), Fmoc-amino acids from Novabiochem (Läufelfingen, Switzerland), and Kaiser test kits from Sigma-Aldrich (Buchs, Switzerland). *N*-Succinimidyl iodoacetate and iodogen tubes were from Pierce Biotechnology (Rockford, IL, USA), Na^125^I (3.7 GBq/mL) from Perkin Elmer (Waltham, MA, USA), and ^111^InCl_3_ (370 MBq/mL) from Mallinckrodt (Petten, The Netherlands). 1,10-Phenanthroline was bought from Merck (Darmstadt, Germany), and all other organic reagents were obtained from Sigma-Aldrich. All reagents were of highest purity available. Cell culture media were from Biochrom AG (Berlin, Germany) and Sigma-Aldrich. Penicillin, streptomycin, vitamins, and non-essential amino acids were bought from Gibco/Invitrogen (Carlsbad, CA, USA) or Sigma-Aldrich.

### Instrumentation

Continuous-flow peptide synthesis was carried out on a Pioneer peptide synthesizer from PerSeptive Biosystems (Framingham, MA, USA). Analytical reverse-phase high-pressure liquid chromatography (RP-HPLC) was performed on a PU-980 system from Jasco (Easton, MD, USA) using a Vydac 218TP54 C18 (5 µm, 4.6 mm × 250 mm) or a Phenomenex Jupiter C18 300 Å (5 µm, 4.6 mm × 250 mm) analytical column. DOTA-NAP-amide-d-Asp-d-Asp was chromatographed with a gradient between solvent A [0.1% trifluoroacetic acid (TFA) in H_2_O] and solvent B (0.1% TFA in 70:30 acetonitrile/H_2_O). The 40-min gradient cycle consisted of the following parts: 95% A (0–2 min), 95–70% A (2–10 min), 70–30% A (10–30 min), 30–5% A (30–34 min), 5% A (34–36 min), 5–95% A (36–38 min), and 95% A (38–40 min); the flow rate was 1 mL/min. UV absorption was recorded at 280 nm using a Jasco UV-1570 detector. DOTA-Phospho-MSH_2-9_ was chromatographed on a Phenomenex Jupiter column using the same gradient, except that solvent A was replaced by 0.02 M ammonium acetate. Mass spectra were determined on a Finnigan LCQ Deca electrospray ion trap mass spectrometry (MS) system.

The purity of the *radioligands* was assessed by RP-HPLC using a dedicated Jasco PU-980 chromatography system equipped with a Spherisorb ODS2/5-μm column and a Radiomatic 500TR LB506C1 γ-detector (Packard, Meriden, CT, USA). Solvent A was 0.1% TFA in H_2_O; solvent B was 0.1% TFA in acetonitrile; the gradient consisted of 96% A (0–2 min), 96–45% A (2–22 min), 45–25% A (22–30 min), 25% A (30–32 min), and 25–96% A (32–34 min); the flow rate was 1.0 mL/min. A cell harvester (Packard) was used to collect cell-bound radioactivity from binding assays on filters. Their radioactivity was measured on a TopCount microplate scintillation counter (Packard). Radioactivity in internalization and biodistribution assays was measured on a Cobra II Auto-Gamma γ-counter (Packard). For bioassays, melanin content in cell culture media of each well was quantified on a Spectra Max 190 microplate reader (Molecular Devices, Menlo Park, CA, USA) using a wavelength of 310 nm.

### Peptide Synthesis

#### General

Continuous-flow solid-phase peptide synthesis was used, combined with Fmoc strategy and the following resins: Flow-compatible Fmoc-PAL-PEG-PS polystyrene resin containing the acid-labile amide linker 5-(4-aminomethyl-3,5-dimethoxyphenoxy)-valeric acid (substitution 0.21 mmol/g) for NAP-amide and DOTA-Phospho-MSH_2-9_, and Fmoc-d-Asp(*t*Bu)-TentaGel S AC resin, a low crosslinked polystyrene matrix resin, for NAP-amide-d-Asp-d-Asp-OH. Amino acid side chains were protected as follows: Trt for Cys and His, *t*-butoxycarbonyl (Boc) for Lys and Trp, *tert*-butyl (*t*Bu) for Asp and d-Asp, and Pbf for Arg. Manual Fmoc deprotection was done in 20% piperidine/DMF for 20 min, followed by a short wash with 20% piperidine/DMF and five washes with DMF; completion of deprotection was assessed by Kaiser test. Cleavage of the peptide from the resin was performed with a solution of 90% TFA, 5% thioanisole, 4.5% H_2_O, and 0.5% 1,2-ethanedithiol. After 2 h, the solution was filtrated, and the peptide precipitated with a 10-fold volume of *t*Bu-methyl ether or diethylether. All reactions and manipulations with DOTA were carried out in acid-treated (1 M HCl, >1 h) glassware.

#### DOTA-NAP-Amide

NAP-amide (13) was assembled as described above. The free *N*-terminus was acetylated at room temperature for 24 h by incubation of the peptide-resin with 2 equivalents of *p*-nitrophenyl acetate, preactivated with hydroxybenzotriazole (HOBt) (1 eq) in DMF for 10 min. The resin was filtrated and washed 5× with DMF and 4× with isopropanol and then submitted to cleavage as described above. The DOTA moiety was coupled to the ε-amino group of the *C*-terminal Lys residue, the peptide conjugate deprotected (90% TFA, 4 h) and purified by RP-HPLC (*t*_R_ = 9.53 min). Calculated monoisotopic mass: 1,485.64/gmol; found: 1,485.65/gmol.

#### DOTA-NAP-d-Asp-d-Asp

**Figure 2:**
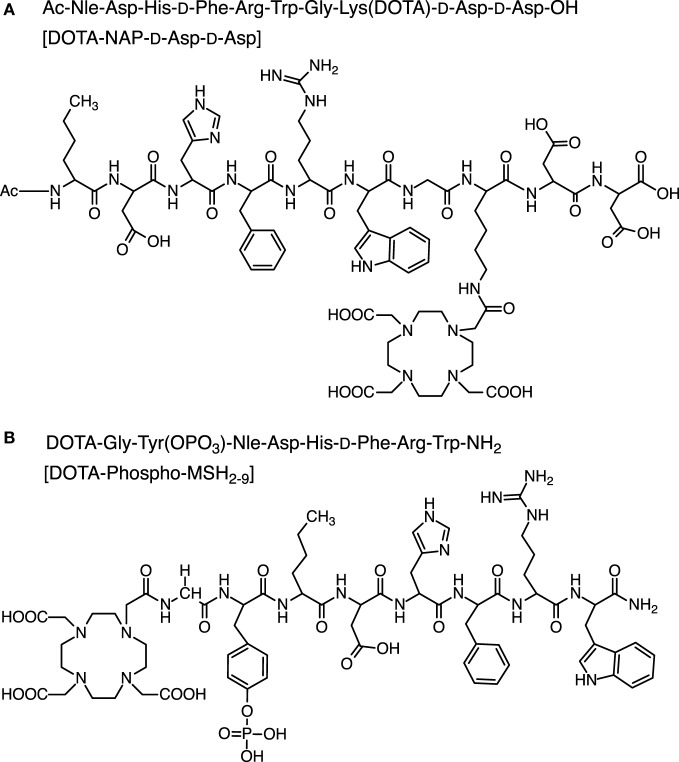
**Chemical structure of DOTA-NAP-d-Asp-d-Asp (A) and DOTA-Phospho-MSH_2-9_ (B)**.

NAP-amide-d-Asp-d-Asp-OH was synthesized, acetylated, and cleaved from the resin as described for NAP-amide (Figure 2A). NAP-amide-d-Asp-d-Asp-OH was purified by HPLC (*t*_R_ = 16.1 min) and lyophilized. Calculated monoisotopic mass: 1,330.40/gmol; found: 1,329.9/gmol. The deprotected peptide (1 eq) was conjugated with DOTA-tris(*t*-butyl ester) (1 eq) and preincubated with HATU (1.2 eq) for 10 min, in the presence of DIPEA (2 eq) using DMF as a solvent. After 1 h at room temperature, half of the initial quantity of preactivated DOTA-tris(*t*-butyl ester) was added to the mixture, and after a total reaction time of 2 h, the peptide was precipitated in ice-cold diethylether. The DOTA moiety was then deprotected by addition of 90% TFA (4 mL per 5 mg of peptide). The mixture was stirred for 4 h, and deprotected DOTA-peptide was precipitated in ice-cold diethylether, resuspended in 10% acetic acid, and purified by RP-HPLC (*t*_R_ = 16.7 min). Calculated monoisotopic mass: 1,716.80/gmol; found: 1,716.79/gmol.

#### DOTA-Phospho-MSH_2-9_

The first amino acid was coupled to the Fmoc-PAL-PEG-PS resin manually; Fmoc-Trp(Boc)-OH (3 eq) was preactivated with HOBt (3 eq + 15%) for 10 min (Figure 2B). This mixture was added to a suspension of the Fmoc-deprotected resin, followed by DIPC (3 eq) for overnight reaction. The resin was washed 5× with DMF, and completion was checked by Kaiser test. The subsequent synthesis steps up to Nle^3^ were done on the peptide synthesizer. After deprotection of the *N*-terminal Fmoc-protecting group, Fmoc-Tyr(PO(OBzl)-OH)-OH (5 eq) was preactivitated with HOBt (1 eq) and then manually coupled to the peptide (1 eq peptide on resin) by addition of TBTU (1 eq) and DIPEA (15 eq). After overnight incubation, the reaction was repeated under the same conditions, and then the Fmoc-protecting group was cleaved. The final amino acid Fmoc-Gly-OH was coupled in the same way. After Fmoc deprotection, DOTA was coupled to the peptide still on the resin by preactivating DOTA (3 eq) with HATU (3 eq) for 10 min and then by adding DIPEA (9 eq) to the resin suspension (1 eq peptide) for overnight reaction. The peptide was simultaneously cleaved from the resin and deprotected by addition of 90% TFA for 4 h and precipitated with ice-cold diethylether. DOTA-Phospho-MSH_2-9_ was finally purified by RP-HPLC (*t*_R_ = 16.5 min). Calculated monoisotopic mass: 1,558.59/gmol; found: 1,558.30/gmol.

### Radiolabeling of Peptides

#### Labeling with ^111^In

Incorporation of ^111^In into DOTA-peptides was performed by the addition of 55.5 MBq of ^111^InCl_3_ to the DOTA-peptides (10 nmol) that had been dissolved in 54 µL acetate buffer (0.4 M, pH 5) containing 2 mg of gentisic acid. Incubation for 10 min at 95°C allowed completion of the reaction. The radiolabeled DOTA-peptides were purified on a small RP cartridge (Sep-Pak C18, Waters) by first washing the column with 0.4 M sodium acetate buffer (pH 7) and then eluting the peptides with ethanol. The purity of the radioligands was assessed by RP-HPLC/γ detection as described above. The specific activity of the radioligand was always >7.4 GBq/μmol.

#### Radioiodination

NDP-MSH (12.14 nmol) was mixed with Na^125^I (37 MBq; Perkin Elmer) in 60 µL phosphate buffer (0.3 M, pH 7.4) in a Iodogen^®^-precoated tube. After a 15-min incubation at room temperature under agitation, the iodination mixture was loaded onto a Sep-Pak C18 cartridge, which was washed consecutively with water and acetic acid (0.5 M). Finally, the peptide was eluted with methanol. The collected fractions containing [^125^I]NDP-MSH were supplemented with dithiothreitol (1.5 mg/mL) and stored at −20°C. Each binding experiment was preceded by an additional purification of the radiotracer by RP-HPLC and subsequent lyophilization from lactose/bovine serum albumin (BSA) (20 mg of each per milliliter of tracer solution).

### Cell Culture

Mouse B16-F1 melanoma cells (39) were cultured in modified Eagle’s medium (MEM) containing 10% heat-inactivated fetal calf serum, 2 mmol/L l-glutamine, 1% non-essential amino acids, 1% vitamin solution, 50 IU/mL penicillin, and 50 µg/mL streptomycin, in a humidified atmosphere consisting of 95% air and 5% CO_2_ at 37°C. For cell expansion or experiments with isolated cells, the B16-F1 cells were detached with 0.02% EDTA in phosphate-buffered saline (PBS) (150 mM, pH 7.2–7.4). The human HBL melanoma cell line (3, 4) was cultured in modified RPMI medium supplemented with 10% heat-inactivated fetal calf serum, 2 mM l-glutamine, 50 IU/mL penicillin, and 50 µg/mL streptomycin in the same conditions as for B16-F1 cells.

### *In Vitro* Binding Assay

Triplicates of 100-µL volumes of B16-F1 or HBL cell suspensions adjusted to 4 × 10^6^/mL were incubated in 96-well U-bottom microplates (Falcon 3077). The binding medium consisted of MEM with Earle’s salts, 0.2% BSA, and 0.3 mM 1,10-phenanthroline. This binding medium was called mouse binding medium (MBM). Triplicates of competitor peptide solution (50 µL) yielding final concentrations ranging from 1 × 10^−6^ to 1 × 10^−12^ M were added. [^125^I]NDP-MSH (50,000 cpm) in 50 µL was finally added to each well. The plates were incubated at 15°C for 3 h for B16-F1 cells and at 37°C for 2 h for HBL cells. The incubation was stopped by covering the plates with ice for 10 min. A cell harvester was used to collect cell-bound radioactivity on filters (Packard Unifilter-96 GF/B). The collected radioactivity was counted on a TopCount scintillation counter (Packard) after addition of 50 µL Microscint-20 scintillation cocktail (Perkin Elmer). The IC_50_ values were calculated with the Prism 6 software (GraphPad Software Inc., San Diego CA, USA).

### *In Vitro* Melanin Assay

The biological activity of the α-MSH derivatives was assessed with an *in situ* melanin assay (40). Briefly, B16-F1 cells (2,500 cells per well in 100 µL) were distributed into 96-well flat-bottom cell culture plates. MEM without phenol red, supplemented with 10% heat-inactivated fetal calf serum, 2 mM l-glutamine, 0.31 mmol/L l-tyrosine, 1% non-essential amino acids, 1% vitamin solution, 50 IU/mL penicillin, and 50 µg/mL streptomycin, was used as culture medium. After overnight incubation at cell culture conditions mentioned above, concentrations of α-MSH derivatives ranging from 1 × 10^−8^ to 1 × 10^−12^ in 100-µL volumes were added (in threefold dilution steps), and the incubation was continued for an additional 72 h. Melanin production was quantified by determining the absorbance at 310 nm in a microplate reader.

### *In Vitro* Internalization Assay

B16-F1 cells were seeded in six-well plates and incubated overnight in MEM at 37°C. For the internalization experiments, MEM was replaced by 1 mL MBM (see above) as internalization buffer. After a 1-h incubation at 37°C, 74 kBq of radioligand (^111^In-labeled peptides) was added, and the plates were incubated for different time periods. Non-specific internalization was determined by addition of 50 µL of a 1 µM α-MSH solution to the incubation mixture. After the desired incubation times, the cells were extensively washed with MBM kept at 37°C to remove excess radioligand. Incubation in 2 mL ice-cold acid buffer (acetate-buffered Hank’s balanced salt solution, pH 5) for 10 min allowed dissociation of surface-bound ligand. After collection of the acid buffer fraction, the cells were rinsed once with cold MBM, and the washings were pooled with the acid buffer fraction. The cells were washed again with MBM kept at 37°C, lysed in a 1% Triton X-100 solution, and finally transferred to tubes for quantification. The radioactivity of all collected fractions was measured in a γ-counter. A counting plate underwent the same treatment as the plate incubated for the longest time, but its cells were detached with 0.02% EDTA in PBS instead of being lysed with the Triton X-100 solution. Cells from three wells were collected, counted, and thus allowed for normalization of the results obtained. Results were expressed as percent of the added dose per million cells.

### *In Vivo* Biodistribution and Stability of the Radioligands in B16-F1 Tumor-Bearing Mice

About 5 × 10^5^ B16-F1 cells were implanted subcutaneously to female B6D2F1 mice (C57BL/6 × DBA/2F1 hybrids; breeding pairs obtained from IFFA-CREDO, France). Seven days later, 185 kBq of ^111^In-labeled ligand in 200 µL PBS/0.1% BSA were injected intravenously into the lateral tail vein of each mouse. Control mice were injected with a mixture of the tracer and 50 µg α-MSH to determine the non-specific uptake of radioligand. The animals were sacrificed 4, 24, and 48 h postinjection, dissected, and the tissues of interest collected, rinsed of excess blood, and weighed. The radioactivity emitted by each organ was measured in a γ-counter to determine the tissue uptake as percentage of the injected dose (ID) per gram of tissue. The total of injected radioactivity per animal was determined by extrapolation from the counts of a standard collected from the injection solution for each animal. Urine samples were collected at 10, 15, and 20 min and 4 h after injection and kept frozen at −80°C until use. Urine (1 vol) was mixed with methanol (2 vol) to precipitate proteins, and the supernatant was analyzed by RP-HPLC/γ-detection, as described in Ref. (12).

### Analysis of Data

Results are expressed as means ± SEM, unless otherwise stated. The statistical evaluation of data was performed using the one- or two-way ANOVA test. When significant overall effects were obtained by ANOVA, multiple comparisons were made with the Bonferroni correction. *P* < 0.05 was considered statistically significant. The area under the curve (AUC) was calculated with the GraphPad Prism 6 software for the indicated period of time, using the mean tissue uptake value at each time point.

## Results

### Peptide Synthesis

All DOTA-peptides were obtained in >99% purity. The synthesis of DOTA-NAP-amide had an overall yield (after RP-HPLC purification) of 15%, DOTA-NAP-d-Asp-d-Asp 8.3%, and DOTA-Phospho-MSH_2-9_ 5.3%. The expected molecular weights were confirmed by MS. The net charges of DOTA-NAP-d-Asp-d-Asp and DOTA-Phospho-MSH_2-9_ at physiological pH are −2 and −1, respectively, calculated on the basis of known pK_a_ values for amino acid residues and functional groups.

### *In Vitro* Receptor-Binding Affinity and Biologic Activity

The binding affinity of the peptides to MC1R was assessed by competition binding assays using [^125^I]-NDP-MSH as radioligand and both murine B16-F1 and human HBL cells. Table 1 summarizes the IC_50_ values obtained for the tested peptide compared to the values of the native ligand α-MSH and the reference peptide DOTA-NAP-amide. DOTA-Phospho-MSH_2-9_ displayed affinities in the nanomolar range on both cell lines. Although the IC_50_ obtained for DOTA-Phospho-MSH_2-9_ with B16-F1 cell line was slightly lower than that of DOTA-NAP-amide (2.32 ± 0.80 vs. 1.38 ± 0.35 nmol/L), the binding affinity with HBL cells was comparable (3.03 ± 0.59 vs. 3.09 ± 1.11 nmol/L). DOTA-Phospho-MSH_2-9_ displayed good α-MSH agonist activity (Table 1), as demonstrated by the induction of melanin synthesis by B16-F1 cells at a dose matching its IC_50_. By contrast, *C*-terminal extension of DOTA-NAP by d-Asp-d-Asp led to a ~10-fold lower MC1R affinity compared to that of DOTA-NAP-amide with both B16-F1 and HBL cells. The biological activity in the melanin assay was also around 11-fold lower than the reference peptide.

**Table 1:** **Receptor-binding potency and biological activity of DOTA-MSH analogs using mouse B16-F1 and human HBL melanoma cells**.

Peptide	Receptor-binding potency IC_50_ (nmol/L)^a^		Biological activity *r*EC_50_ [α-melanocyte-stimulating hormone (α-MSH) = 1]^b^
	B16-F1	HBL	B16-F1
α-MSH	1.50 ± 0.14	1.91 ± 0.26	1
DOTA-NAP-amide	1.38 ± 0.35	3.09 ± 1.11	0.66 ± 0.35
DOTA-NAP-d-Asp-d-Asp	19.67 ± 4.48^c^	29.80 ± 7.96^c^	7.66 ± 0.33^c^
DOTA-Phospho-MSH_2-9_	2.32 ± 0.80	3.03 ± 0.59	0.85 ± 0.11

*^a^IC_50_ values are the concentrations inducing half-maximal binding inhibition of the MSH analogs and were determined in competition binding experiments using [^125^I]-NDP-MSH as radioligand and mouse B16-F1 and human HBL human melanoma cells (*n* = 3–20)*.

*^b^*r*EC_50_ values represent the relative concentrations compared to α-MSH (=1) inducing half-maximal melanin production by B16-F1 cells (*n* = 3–13)*.

*^c^P < 0.05 vs. DOTA-NAP-amide*.

### Internalization

**Figure 3:**
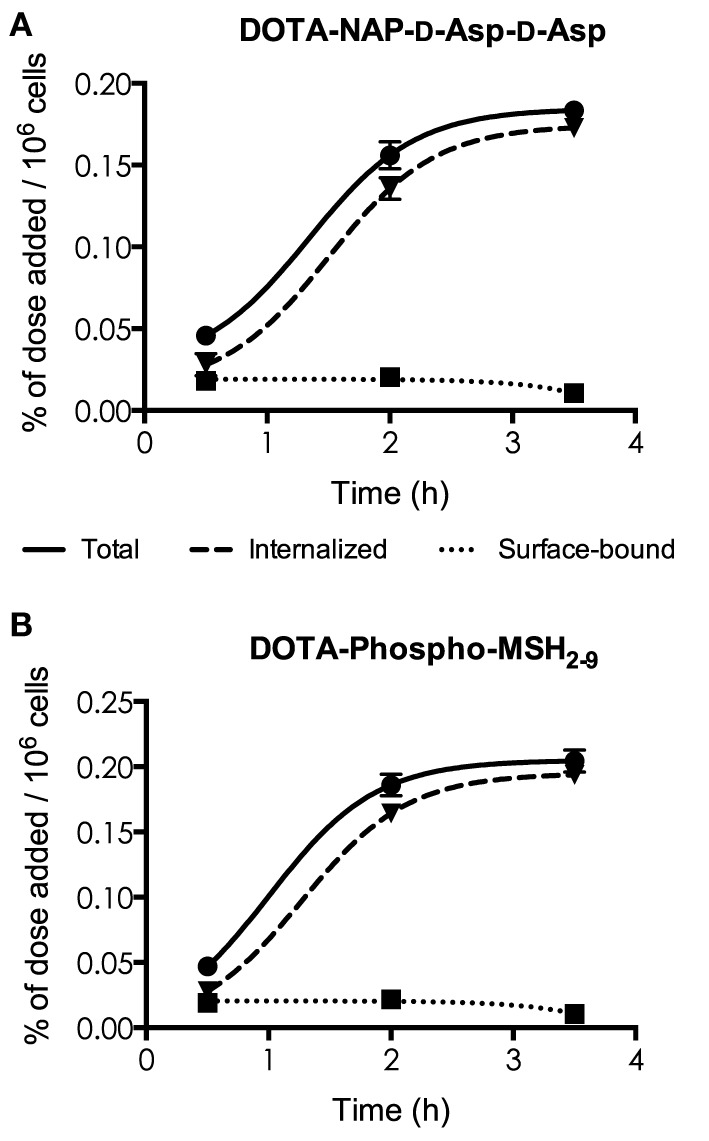
**Determination of internalization of [^111^In] DOTA-NAP-d-Asp-d-Asp (A) and [^111^In]DOTA-Phospho-MSH_2-9_ (B) by cultured B16-F1 cells exposed to the peptides at 37°C for 0.5, 2, and 3.5 h**. Surface-bound radioligand was released by an acid buffer wash, and internalized radioligand was determined by lysing cells with detergent. Results are expressed in percent of the added dose per million cells.

[^111^In]DOTA-NAP-d-Asp-d-Asp and [^111^In]DOTA-Phospho-MSH_2-9_ exhibited excellent internalization profiles when studied *in vitro* with cultured B16-F1 cells (Figure 3). The plateau phase was not quite reached by the first peptide after 3.5 h, probably because of the lower receptor affinity; this may predict lower *in vivo* tumor uptake. By contrast, the plateau phase for [^111^In]DOTA-Phospho-MSH_2-9_ was almost complete after 3.5 h, indicating that maximal internalization of the peptide was reached after 4 h, the first test point with *in vivo* biodistribution studies. It appears that MC1R internalization was not altered by the negatively charged ligands.

### Biodistribution in Tumor-Bearing Mice

Tissue distributions in B16-F1 melanoma-bearing mice of [^111^In]DOTA-NAP-d-Asp-d-Asp and [^111^In]DOTA-Phospho-MSH_2-9_ were compared with the reference peptide [^111^In]DOTA-NAP-amide (Table 2). Tissues, including melanoma tumors, were collected 4, 24, and 48 after injection of the radiopeptides. Clearance from the blood was faster for the two new radiopeptides compared with the reference peptide as almost no radioactivity could be detected after 4 h. Kidney uptake of [^111^In]DOTA-NAP-d-Asp-d-Asp was 5.95 ± 0.85 %ID/g 4 h after injection, i.e., the uptake was 25% higher than that of [^111^In]DOTA-NAP-amide (4.77 ± 0.26 %ID/g), which was not expected. After 24 h, the radioactive load was even 70% higher than with the reference peptide. On the other hand, owing to the lower receptor affinity of [^111^In]DOTA-NAP-d-Asp-d-Asp, it was anticipated that tumor uptake (1.93 ± 0.11 %ID/g after 4 h) was lower than that of [^111^In]DOTA-NAP-amide (7.77 ± 0.35 %ID/g). Other organs did not substantially accumulate the radiopeptide.

**Table 2:** **Tissue biodistribution of ^111^In-labeled DOTA-NAP-amide, DOTA-NAP-d-Asp-d-Asp, and DOTA-Phospho-MSH_2-9_ at 4, 24, and 48 h after injection into tumor-bearing mice**.

Organ	Time (h)	Uptake (%ID/g of tissue **±** SEM)^a^		
		DOTA-NAP-amide	DOTA-NAP-d-Asp-d-Asp	DOTA-Phospho-MSH_2-9_
Blood	4	0.09 ± 0.02	0.01 ± 0.00	0.02 ± 0.00
	24	0.02 ± 0.00	0.01 ± 0.00	0.01 ± 0.00
	48	0.00 ± 0.00	0.01 ± 0.00	0.00 ± 0.00
Tumor	4	7.77 ± 0.35	1.93 ± 0.11	7.33 ± 0.47
	24	2.32 ± 0.15	0.63 ± 0.03	2.92 ± 0.12
	48	1.41 ± 0.12	0.23 ± 0.02	1.21 ± 0.18
Stomach	4	0.09 ± 0.01	0.11 ± 0.00	0.17 ± 0.08
	24	0.12 ± 0.02	0.03 ± 0.00	0.16 ± 0.02
	48	0.11 ± 0.05	0.02 ± 0.00	0.07 ± 0.00
Kidney	4	4.77 ± 0.26	5.95 ± 0.85	2.68 ± 0.18
	24	2.41 ± 0.20	4.09 ± 0.16	1.88 ± 0.11
	48	1.55 ± 0.07	2.02 ± 0.08	1.04 ± 0.07
Liver	4	0.34 ± 0.05	0.10 ± 0.00	0.20 ± 0.01
	24	0.31 ± 0.02	0.09 ± 0.00	0.16 ± 0.02
	48	0.27 ± 0.07	0.07 ± 0.00	0.12 ± 0.01
Spleen	4	0.14 ± 0.01	0.07 ± 0.00	0.11 ± 0.01
	24	0.11 ± 0.01	0.07 ± 0.00	0.10 ± 0.01
	48	0.10 ± 0.01	0.07 ± 0.00	0.09 ± 0.01
Lung	4	0.08 ± 0.01	0.06 ± 0.00	0.07 ± 0.02
	24	0.05 ± 0.01	0.04 ± 0.00	0.04 ± 0.00
	48	0.03 ± 0.00	0.03 ± 0.00	0.03 ± 0.00
Small intestines	4	0.07 ± 0.01	0.05 ± 0.01	0.11 ± 0.03
	24	0.08 ± 0.01	0.04 ± 0.01	0.06 ± 0.00
	48	0.05 ± 0.01	0.03 ± 0.00	0.06 ± 0.00
Pancreas	4	0.04 ± 0.00	0.03 ± 0.00	0.05 ± 0.01
	24	0.03 ± 0.00	0.02 ± 0.00	0.03 ± 0.00
	48	0.02 ± 0.00	0.03 ± 0.00	0.03 ± 0.00
Heart	4	0.05 ± 0.01	0.03 ± 0.00	0.04 ± 0.00
	24	0.03 ± 0.00	0.03 ± 0.00	0.03 ± 0.00
	48	0.01 ± 0.00	0.03 ± 0.00	0.03 ± 0.00
Bone	4	0.11 ± 0.02	0.07 ± 0.01	0.08 ± 0.01
	24	0.14 ± 0.02	0.06 ± 0.01	0.11 ± 0.02
	48	0.05 ± 0.01	0.07 ± 0.01	0.06 ± 0.01
Muscle	4	0.05 ± 0.01	0.02 ± 0.00	0.02 ± 0.00
	24	0.02 ± 0.00	0.02 ± 0.00	0.02 ± 0.00
	48	0.01 ± 0.00	0.03 ± 0.00	0.02 ± 0.00
Skin	4	–	0.06 ± 0.01	0.12 ± 0.03
	24	–	0.06 ± 0.00	0.07 ± 0.02
	48	–	0.04 ± 0.00	0.08 ± 0.02

*^a^The values are the means of three experiments each of which consisted of n = 5 animals per compound*.

**Figure 4:**
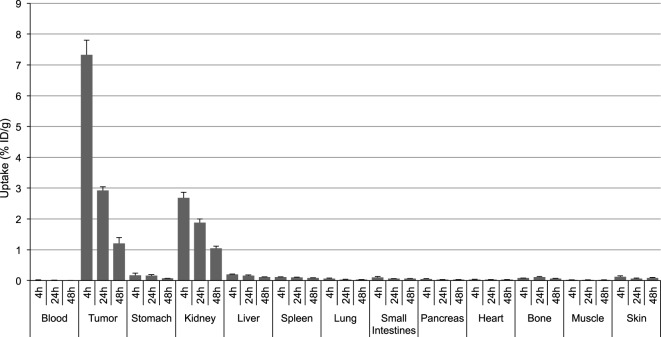
**Tissue distribution of [^111^In]DOTA-Phospho-MSH_2-9_ in B16-F1 melanoma tumor-bearing mice at 4, 24, and 48 h postinjection**. Results are expressed as percent of injected dose per g of tissue (%ID/g; means ± SEM; *n* = 3 experiments).

[^111^In]DOTA-Phospho-MSH_2-9_ displayed more promising data: after 4 h, tumor uptake was 7.33 ± 0.47 %ID/g, i.e., almost identical with that of [^111^In]DOTA-NAP-amide, and kidney uptake was 2.68 ± 0.18 %ID/g, which corresponds to 56% of that of the reference peptide (Table 2). Non-specific uptake by other organs was very low and did not exceed the values found with [^111^In]DOTA-NAP-amide (Figure 4), thus excluding an altered excretion pathway. Coinjection of 50 µg of α-MSH together with the radiopeptide significantly blocked melanoma uptake and confirmed MC1R-mediated internalization (data not shown). The retention of radioactivity by the tumor was decreased to 40% at 24 h postinjection and to 15% at 48 h postinjection. These data are similar to those found with other MSH radiopeptides. On the other hand, clearance from the kidney appeared to be slightly slower. The tumor-to-kidney ratios calculated for 4, 24, and 48 h were 2.75, 1.55, and 1.16, respectively (Table 3). This results in an AUC (4–48 h) of 1.81 compared with 1.17 for [^111^In]DOTA-NAP-amide.

**Table 3:** **Tumor-to-kidney ratios for tissue uptake of [^111^In]DOTA-NAP-amide, [^111^In]DOTA-NAP-d-Asp-d-Asp, and [^111^In]DOTA-Phospho-MSH_2-9_ after 4, 24, and 48 h postinjection**.

Peptide	Tumor-to-kidney ratios^a^		
	4 h	24 h	48 h
[^111^In]DOTA-NAP-amide	1.63	0.96	0.91
[^111^In]DOTA-NAP-d-Asp-d-Asp	0.32	0.15	0.11
[^111^In]DOTA-Phospho-MSH_2-9_	2.74	1.55	1.16

*^a^Ratios are presented as quotient between the means of tumor uptake divided by means of kidney uptake (Table 2)*.

### Uptake by the Kidneys and Liver in Relation to the Overall Net Charge of MSH Analogs

**Figure 5:**
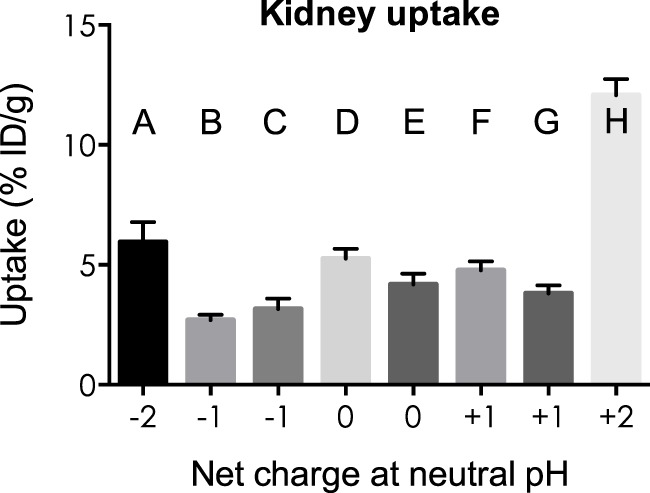
**Uptake by the kidneys of eight different ^111^In-labeled DOTA-MSH peptides with net charges ranging from −2 to +2**. For structures of peptides A–H, see Table 4. Results are expressed as percent of injected dose per gram of tissue (%ID/g; means ± SEM; *n* = 3).

**Figure 6:**
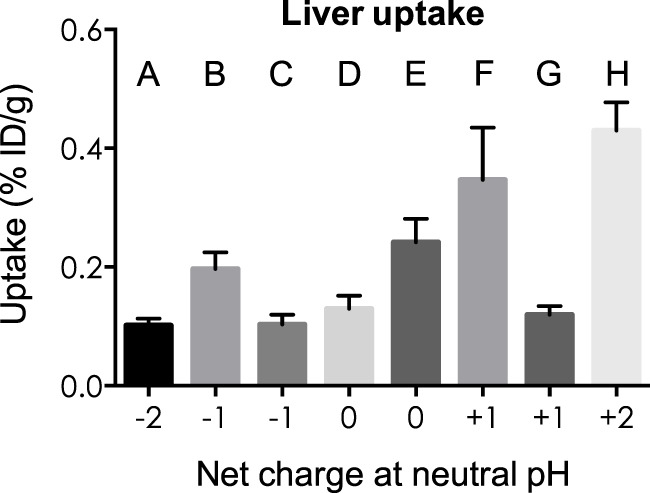
**Uptake by the liver of eight different ^111^In-labeled DOTA-MSH peptides with net charges ranging from −2 to +2**. For structures of peptides A–H, see Table 4. Results are expressed as percent of injected dose per gram of tissue (%ID/g; means ± SEM; *n* = 3).

Kidney and liver uptake of [^111^In]DOTA-NAP-d-Asp-d-Asp, [^111^In]DOTA-Phospho-MSH_2-9_, and [^111^In]DOTA-NAP-amide (reference peptide) was compared with that of five previously published peptides (14) that altogether cover an overall net charge ranging between +2 and −2 (Table 4). As shown in Figure 5, kidney uptake is lowest for net charge −1; it is considerably increased for −2 and +2 and moderately for +1 or 0. Of the two peptides with a net charge of −1, [^111^In]DOTA-Phospho-MSH_2-9_ showed a slightly lower kidney uptake than [^111^In-DOTA-Nle^4^,Asp^5^,d-Phe^7^,Lys^11^(Suc)]-α-MSH_4-11_-carboxylate, indicating that incorporating the negative charges in the *N*-terminal region is more advantageous than in the *C*-terminal region. In addition, the AUC (4–48 h) for [^111^In]DOTA-Phospho-MSH_2-9_ was 1.81, i.e., the tumor-to-kidney ratio was threefold higher than that of [^111^In-DOTA-Nle^4^,Asp^5^,d-Phe^7^,Lys^11^(Suc)]-α-MSH_4-11_-carboxylate exhibiting an AUC (4–48 h) of 0.62 (14). With respect to the liver, non-specific uptake does not follow the same pattern as outlined for the kidneys (Figure 6). Other factors such as differences in lipophilicity may play a more important role. It should be stressed, however, that liver uptake is about 20-fold lower than that reported for the kidneys and therefore of minor importance for these peptides.

**Table 4:** **Comparison of DOTA-NAP-d-Asp-d-Asp and DOTA-Phospho-MSH_2-9_ with different analogs of MSH-NAP-amide containing modifications at the *C*-terminal or *N*-terminal end or in the N^ε^-lysine side chain that yield different overall net charges**.

Peptide		*N*-terminal	*C*-terminal	N^ε^-Lys	Net charge	Reference
A	{[Ac-Nle^4^,Asp^5^,d-Phe^7^,Lys^11^(DOTA)]-α-MSH_4-11_}-d-Asp-d-Asp-OH	Ac	d-Asp-d-Asp	DOTA	−2	
B	[DOTA-Gly^2^,Tyr(P)^3^,Nle^4^,Asp^5^,d-Phe^7^]-α-MSH_2-9_ [DOTA-Phospho-MSH_2-9_]	DOTA	Trp-amide	–	−1	
C	[DOTA-Nle^4^,Asp^5^,d-Phe^7^,Lys^11^(Suc)]-α-MSH_4-11_-carboxylate	DOTA	OH	Suc	−1	(14)
D	[DOTA-Nle^4^,Asp^5^,d-Phe^7^,Lys^11^(Suc)]-α-MSH_4-11_	DOTA	Amide	Suc	0	(14)
E	[Ac-Nle^4^,Asp^5^,d-Phe^7^,Lys^11^(DOTA)]-α-MSH_4-11_-carboxylate	Ac	OH	DOTA	0	(14)
F	[Ac-Nle^4^,Asp^5^,d-Phe^7^,Lys^11^(DOTA)]-α-MSH_4-11_ [DOTA-NAP-amide]	Ac	Amide	DOTA	+1	(13)
G	[DOTA-Nle^4^,Asp^5^,d-Phe^7^,Lys^11^(Ac)]-α-MSH_4-11_	DOTA	Amide	Ac	+1	(14)
H	[DOTA-Nle^4^,Asp^5^,d-Phe^7^]-α-MSH_4-11_	DOTA	Amide	H	+2	(14)

## Discussion

The net charge of radiolabeled peptides appears to be a crucial factor with respect to excretion *via* the urinary tract. The negatively charged surface of tubular cells of the kidneys involves repulsing electrostatic effects on negatively charged molecules passing in the luminal part of the proximal tubules, thus reducing their (re-)uptake (30, 41). As already mentioned, this effect can be further enhanced by the presence of positively charged amino acids such as Lys and Arg (42). An earlier study in our laboratory comparing differently charged DOTA-NAP-amide type of peptides demonstrated that [^111^In-DOTA-Nle^4^,Asp^5^,d-Phe^7^,Lys^11^(Suc)]-α-MSH_4-11_-carboxylate with a net charge of −1 exhibited the lowest kidney uptake but was nevertheless not an alternative to [^111^In]DOTA-NAP-amide because of the lower MC1R affinity and reduced uptake by melanoma tumors (14). With the new peptide DOTA-NAP-d-Asp-d-Asp (net charge −2), the question of a potentially positive effect of an additional negative charge at the *C*-terminus was addressed. *In vitro*, this peptide showed rapid internalization by cultured B16-F1 melanoma cells despite an ~10-fold reduced MC1R affinity compared to DOTA-NAP-amide. *In vivo*, uptake by melanoma tumors was ~4-fold lower, but uptake/retention by the kidneys was markedly increased. These findings would contradict the expectation of an even higher electrostatic repulsion on the surface of tubular cells. It is possible that once internalized, the fragmentation of the *C*-terminal region of DOTA-NAP-d-Asp-d-Asp is retarded by the d-Asp residues and excretion of Lys([^111^In]DOTA), a metabolite of internalized [^111^In]DOTA-NAP-amide occurring 4 h after injection (Froidevaux and Eberle, unpublished), is slower than for the reference compound. In summary, addition of a total of three negative charges to the *C*-terminus of DOTA-NAP-amide, yielding a net charge of −2 of the molecule, impairs its pharmacokinetic properties.

To find an alternative to [DOTA-Nle^4^,Asp^5^,d-Phe^7^,Lys^11^(Suc)]-α-MSH_4-11_-carboxylate with a net charge of −1 but higher tumor uptake, we aimed at incorporating the negative charges in the *N*-terminal region of the molecule. To this end, DOTA-Phospho-MSH_2-9_ was prepared, which contains a phosphate group in the phenolic ring of Tyr^2^ of the MSH_2-9_ peptide fragment. This peptide exhibited almost the same *in vitro* characteristics as the reference compound DOTA-NAP-amide. In particular, [^111^In]DOTA-Phospho-MSH_2-9_ was very rapidly internalized by cultured B16-F1 cells and reached the plateau after 3.5 h. *In vivo*, tumor uptake was about as high as for the reference compound but kidney uptake was markedly lower leading to the most favorable tumor-to-kidney ratio of a linear DOTA-MSH radiopeptide reported to date. All other compounds with net charges from +2 to 0 displayed less favorable tumor-to-kidney ratios. In conclusion, linear DOTA-MSH peptides preferably have an overall net charge of −1, and the additional negative charges are incorporated in the *N*-terminal region.

While the experimental work with the two new ^111^In-labeled MSH radiopeptides described here was already completed, the group of Miao and collaborators published several new lactam-bridged cyclized MSH analogs containing DOTA and NOTA chelators (for ^111^In, ^67/68^Ga, ^90^Y) or MAG_3_ and HYNIC chelators (for ^99m^Tc, ^188^Re) for which they found considerably superior biodistribution data (43–46) compared to those with previously published cyclic MSH analogs. These novel cyclic MSH analogs may even be superior to DOTA-Phospho-MSH_2-9_, but this would have to be examined in a comparative study under the same experimental conditions with DOTA-Phospho-MSH_2-9_ to define a potentially even more attractive novel lead compound.

## Ethics Statement

All animal experiments were performed in compliance with Swiss animal welfare regulations and were ethically approved by the Ethics Committee for Animal Experimentation of the University Hospital Basel, followed by review and approval by the Cantonal Commission for Animal Experimentation of Basel. No human data were used in this study.

## Author Contributions

J-PB and AE contributed substantially to the conception and design of the work, the acquisition, analysis and interpretation of data, and the drafting of the work.

## Conflict of Interest Statement

The authors declare that the research was conducted in the absence of any commercial or financial relationships that could be construed as a potential conflict of interest.

## Abbreviations

AUC, area under the curve (quantification during a time interval); Boc, *t*-butoxycarbonyl; *t*Bu, *tert*-butyl; DIPEA, *N*,*N*′-diisopropylethylamine; DIPC, *N*,*N*′-diisopropyl-carbodiimde; DOTA, 1,4,7,10-tetraazacyclododecane-1,4,7,10-tetra-acetic acid; DTPA, diethylenetriaminepentaacetic acid; Fmoc, fluorenylmethoxycarbonyl; HATU, 0-(7-azabenzotriazole-1-yl)-1,1,3,3-tetramethyluronium hexafluorophosphate; HOBt, hydroxybenzotriazole; ID, injected dose; MBM, mouse binding medium; MC1R, melanocortin type 1 receptor; α-MSH, α-melanocyte-stimulating hormone; MS, mass spectrometry; NOTA, 1,4,7-triazacyclononane-1,4,7-triacetic acid; PAL, 5-(4-aminomethyl-3,5-dimethoxyphenoxy)-valeric acid; Pbf, 2,2,4,6,7-pentamethyldihydrobenzofuran-5-sulfonyl; PBS, phosphate-buffered saline; PEG-PS, poly-ethyleneglycol-polystyrene graft; RP-HPLC, reverse-phase high-pressure liquid chromatography; Suc, succinyl; TBTU, *N,N,N′,N′*-tetramethyl-*O*-(benzotriazol-1-yl)uronium tetrafluoroborate; TFA, trifluoroacetic acid; Trt, trityl.
